# Risk Factors for Different Types of Pregnancy Losses: Analysis of 15,210 Pregnancies After Embryo Transfer

**DOI:** 10.3389/fendo.2021.683236

**Published:** 2021-06-25

**Authors:** Ai-Min Yang, Xiuhua Xu, Yan Han, Jian-Jun Wei, Gui-Min Hao, Na Cui, Zhi-Ming Zhao, Wei Wang, Xianghua Huang

**Affiliations:** ^1^ Department of Gynecology and Obstetrics, Second Hospital of Hebei Medical University, Shijiazhuang, China; ^2^ Department of Reproductive Medicine, Second Hospital of Hebei Medical University, Shijiazhuang, China; ^3^ Cardiovascular Platform, Institute of Health and Disease, Hebei Medical University, Shijiazhuang, China; ^4^ Department of Pathology, Northwestern University School of Medicine, Chicago, IL, United States

**Keywords:** pregnancy loss, frozen-thawed embryo transfer, polycystic ovary syndrome, thickness of endometrium, obesity

## Abstract

**Objective:**

To evaluate the risk factors for different types of pregnancy losses after embryo transfer (ET).

**Design:**

Retrospective cohort study.

**Setting:**

Reproductive medicine center.

**Participants:**

A total of 15,210 pregnancies after fresh and frozen-thawed embryo transfer between January 2014 and June 2019.

**Main Outcome Measures:**

The primary outcome was pregnancy loss (PL) throughout the entire pregnancy. Secondary outcomes were non-visualized PL, early miscarriage, late miscarriage, and stillbirth.

**Methods:**

The effect of patients’ baseline characteristics and IVF/ICSI cycle-specific factors on the risk of PL after fresh and frozen-thawed ET was determined by multivariate logistic regression analysis.

**Results:**

Compared to women under 35 years old, those between 35 and 40 had an increased risk of early miscarriage [odds ratio (OR) 1.49, 95% confidence interval (CI) 1.22-1.83], while those after 40 appeared to have an increased risk of both early miscarriage (OR 3.82, 95% CI 2.65-5.51) and late miscarriage (OR 2.79, 95% CI 1.64-4.77). Overweight patients were observed to have a higher risk of late miscarriage (OR 1.38, 95% CI 1.16-1.65), while obese patients showed a higher risk of both early miscarriage (OR 1.47, 95% CI 1.14-1.91) and late miscarriage (OR 1.80, 95% CI 1.33-2.44). Polycystic ovary syndrome (PCOS) was an independent risk factor for late miscarriage (OR 1.58, 95% CI 1.28-1.96), and the detrimental effect of PCOS was independent of obesity status. Women with uterine factors had a higher risk of early miscarriage (OR 1.77 (95% CI 1.32-2.38) than women without uterine factors. A negative correlation was observed between the thickness of the endometrium and PL (OR 0.95 95% CI 0.92-0.97). There was an increased risk of PL after frozen-thawed ET versus fresh ET (OR 1.12, 95% CI 1.01-1.24). Women who transferred ≥2 embryos showed lower risk of overall PL than women who transferred a single embryo, with adjusted ORs ranged from 0.57~0.94. However, women who transferred three embryos demonstrated a higher risk of late miscarriage than women who transferred a single embryo (OR 2.23, 95% CI 1.36-3.66).

**Conclusions:**

Patients with uterine factors demonstrated higher risk of early miscarriage and stillbirth. Being overweight, PCOS, and transferring three embryos was associated with late miscarriage. Being aged 40 and over, obese, and using frozen embryo transfer was associated with early and late miscarriage.

## Introduction

Nowadays, assisted reproductive technology (ART) has become a common technique for infertility treatment worldwide. Although the clinical pregnancy rate has gradually improved over the past decades, the live birth rate is still low, only 38.1% (1). Pregnancy loss (PL) is the spontaneous demise of an embryo throughout the entire pregnancy (2). It includes non-visualized PL and clinical pregnancy losses (3). PL significantly threatens the rate of live birth delivery. Studies on patients conceiving through ART showed that they were confronted with even more of a probability of PL than naturally conceiving women (1, 4, 5). Additionally, PL is associated with physical and emotional trauma (6), particularly in the setting of highly desired pregnancy achieved *via* ART. Therefore, it is of great significance to estimate the risk factors for PL in order to assess the overall effectiveness of *in vitro* fertilization/intra-cytoplasmic sperm injection-embryo transfer (IVF/ICSI-ET) and improve patient counselling before the initiation of treatments.

Previous studies have identified several risk factors for PL. Clinical studies have confirmed that miscarriage rate increased with maternal age increase, especially after 35 (7, 8). A large retrospective cohort study from Provost et al. showed that pregnancy outcomes were most favorable in cohorts with low or normal weight but progressively worsened as BMI increased in fresh IVF cycles (9). Polycystic ovary syndrome (PCOS) patients were confronted with a higher risk of PL than other infertile populations who underwent fresh embryo transfer (ET) (10, 11). However, few studies had explored the correlation between maternal age, maternal BMI, PCOS, and PL after frozen ET. Furthermore, studies had concluded that the thickness of the endometrium was strongly associated with pregnancy outcomes of IVF/ICSI treatment (3), but the optimal threshold of endometrial thickness with a minimal risk of PL has not yet been established. In addition, women who underwent IVF/ICSI treatment may have specific risk factors for miscarriage, including inadequate ovarian reserve (12, 13) and ovulatory dysfunction (14).

Patients conceiving through ART are more closely monitored than naturally conceiving women, which allows for more detailed evaluation of PL-associated risk factors. Although many studies have assessed risk factors for early miscarriage in ART pregnancies (1, 15–17), sparse large scale studies have explored the risk factors throughout the entire pregnancy and distinguished risk factors for different types of PL. However, risk factors could be very different among non-visualized PL, early miscarriage, late miscarriage, and stillbirth. Many studies only analyzed risk factors for PL after fresh or frozen cycles, whereas taking PL for the whole IVF/ICSI treatment into consideration is essential for a more appropriate evaluation. Herein, the objective of this retrospective cohort study was to present an overall outcome of IVF/ICSI-ET treatment and analyze the risk factors for different types of PL. This study could be useful in terms of identification of patients at high risk of PL and may shed light on the modification of ART protocols to minimize the risk of PL.

## Materials and Methods

### Study Population and Design

All the information of patients who underwent ART in our department was stored in a computerized database. This retrospective cohort study followed the Strengthening the Reporting of Observational Studies in Epidemiology (STROBE) guidelines. The study was approved by the ethics committee of the Second Hospital of Hebei Medical University, and written informed consent was obtained from all participants. All participants allowed the use of their medical record data for research anonymously. Patients were aged between 20 and 45 years, and all had indications for IVF/ICSI-ET. Couples’ own gametes were used in all included cycles. The exclusion criteria included treatment with pre-implantation genetic testing (PGT) or for fertility preservation.

PCOS was defined as the presence of menstrual disturbance combined with either hyperandrogenism (hirsutism or hyperandrogenemia) or a polycystic ovary on ultrasonography (defined as either an ovary that contains ≥12 antral follicles or ovarian volume >10 cm^3^), with the exclusion of other causes of hyperandrogenism and ovulation dysfunction (18). Criteria for diagnosing endometriosis were based on a structured process involving the combination of patient interviews, clinical examination, and imaging. Patients were diagnosed with endometriosis if patients met the following criteria combined with typical clinical symptoms and signs (19): Transvaginal ultrasound or MRI-diagnosed superficial peritoneal lesions, ovarian, or pelvic endometriomas and adenomyosis; previous laparoscopic or histologically diagnosed endometriosis lesions. Uterine factors included congenital uterine abnormalities arising from alterations during embryonic development (arcuate, bicornuate, didelphic, septate, T-shaped, and unicornuate anomalies) and acquired uterine malformations (intrauterine adhesions, fibroids, and endometrial polyps) (20). Criteria and classification for congenital uterus malformations were according to the 2013 ESHRE/ESGE consensus on the classification of female genital tract congenital anomalies (21). Patients with male factors were divided into subgroups according to the WHO Laboratory Manual for the Examination and Processing of Human Semen - 5th Edition and diagnosis of obstructive azoospermia. BMI was calculated by the following formula: BMI = weight/height^2^ (kg/m^2^). Primary infertility was defined as the inability to achieve a clinical pregnancy after 12 months of unprotected and regular sexual intercourse when a patient has never conceived, while secondary infertility was the incapability to conceive in a patient who has had at least one successful clinical pregnancy previously. The thickness of the endometrium was measured on the oocyte maturation trigger day in fresh cycles and the ultimate endometrial thickness on the day prior to the start of progesterone administration or the day when the intrinsic LH surge commenced was used in frozen cycles.

### IVF/ICSI-ET Treatment Procedures

Controlled ovarian stimulation (COS) was performed according to the Gonadotrophin-releasing hormone agonist (GnRH) antagonist protocol, luteal phase GnRH agonist protocol, or early-follicular phase GnRHa long protocol in most conditions. The GnRH antagonist protocol was the first-line choice for high and low responders. For normal responders, all the three protocols could be used. For ovarian stimulation, recombinant FSH (follicle-stimulating hormone) and/or urinary FSH were used. Follicular growth was monitored by ultrasound and as soon as at least three of the dominant follicles were ≥18 mm in diameter in the long protocol and ≥3 follicles reached ≥17 mm in the GnRHant protocol, 250 μg of recombinant human chorionic gonadotropin (hCG) was given. Oocyte retrieval was performed 36~38 h after hCG administration. The oocytes were inseminated approximately 4 to 6 h after oocyte retrieval by a conventional method or intracytoplasmic sperm injection. For the scoring of cleavage stage embryos, we employed the criteria which focused on the number and regularity of blastomeres as well as the percentage fragmentation (22), while blastocyst stage embryos were graded according to Gardner et al. (23). All the patients transferred a rating of good or fair embryos according to the Istanbul consensus scoring system for embryos (24). Fresh embryo transfer was performed on day 3~6 after oocyte retrieval. The luteal phase support was performed by vaginal administration of progesterone gel (Crinone, Merck Serono, Watford, UK) starting from the morning of oocyte retrieval day until 14 days after ET.

Natural cycle, induced ovulation cycle, hormone replacement therapy (HRT), and downregulation HRT cycle were used in endometrial preparation for frozen-thawed ET. For the natural cycle and letrozole stimulation protocol, ovulation was monitored by ultrasound. Vitrified-warmed embryos were transferred 3~6 days after ovulation, according to the stages of embryos. Oral dydrogesterone (Duphaston, Abbott, OLST, Netherlands) at 10 mg bid was administered for luteal support. For the HRT protocol, oral oestradiol (Progynova, Delpharm Lille, Lys-Lez-Lannoy, France) 4~8 mg daily or 17β-E_2_ (Fematon, Abbott Healthcare Products) 2~4mg daily was administrated on day 3~5 of the menstrual cycle. Vaginal progesterone gel (Crinone, Merck Serono, Watford, UK) at 90 mg once a day and oral dydrogesterone at a dose of 10 mg three times a day were administrated for luteal support, vitrified–warmed embryos were transferred 3~6 days after the initiation of progesterone administration according to the stages of embryos. If pregnancy was achieved, oral oestradiol was continued until 6 weeks of gestation, while vaginal progesterone gel and oral dydrogesterone were continued until 8 weeks of gestation.

### Pregnancy Assessment

The pregnancy outcomes were obtained from the follow-up database. Pregnancy was defined as serum hCG level ≥5.3mIU/mL or positive urine hCG test 12~14 days after ET. If β-hCG levels were 5.3~10 mIU/mL, blood samples were retested to confirm the results. Clinical pregnancy was confirmed with transvaginal ultrasound visualization of an intrauterine gestational sac 4~5 weeks after ET. Live birth was defined as the birth of at least one living child. PL was defined as the situation when patients had positive hCG but failed to result in any live birth with the exception of ectopic pregnancy and medically induced abortion, because ectopic pregnancy and medically induced abortion are due to different etiology from PL. PL was classified into three subtypes, including non-visualized PL, miscarriage, and stillbirth. This definition of PL reflects the probability of a patient with at least one live birth delivery. Non-visualized PL was defined as any pregnancy, which had not been confirmed ultrasonically or histologically (2). Early miscarriage was defined as uterine PL at <12 gestational weeks, late miscarriage was defined as uterine PL between 12 and 24 gestational weeks, and stillbirth was defined as a baby born dead at 28 weeks of gestation or more, or with a birth weight of ≥1000 g, or a body length of ≥35 cm (25). The live birth rate (LBR) = live-birth deliveries/all studied pregnancies and PL rate = pregnancy losses/all studied pregnancies.

Patients who had ultrasound visualization of at least one gestational sac 3~4 weeks after positive hCG test, will be followed up at 11~13, 14~16, 22~28, and 32~40 weeks of gestation about their antenatal records. The delivery report of each patient was requested shortly after delivery from the general practitioner of the patient to obtain pregnancy data from the patient as well as perinatal data from the child. For patients who had PL, the time to event (gestational age at PL) was calculated as the date of PL minus the date of the theoretical last menstrual period (17 days prior to the date of cleavage-stage embryos transferred and 19 days prior to the date of blastocysts transferred). According to the follow-up data, all PL cycles were divided into non-visualized PL, early miscarriage, late miscarriage, and stillbirth.

### Statistical Methods

The retrospective nature of the study predetermines the sample size. The patients’ baseline characteristics, treatment characteristics, and outcome data were described using frequencies with percentages, or means with standard deviations, as appropriate. The incidence of PL by baseline characteristics and treatment characteristic subgroups were analyzed by the Chi-square test. Univariate and multivariate logistic regression analyses were performed to evaluate the association between the variables and PL. To further determine the association between continuous variables and PL rate, we applied a two-piecewise linear regression model to examine the threshold effect of maternal age, maternal BMI, and thickness of the endometrium on PL rate by using a smoothing function. The threshold level (i.e., turning point) was determined using trial and error, including the selection of turning points along with a pre-defined interval and then choosing the turning point that gave the maximum model likelihood. Lastly, multivariate logistic regression analyses were used to assess the association between the variables and different types of PL (non-visualized PL, early miscarriage, late miscarriage, and stillbirth) across all pregnancies, and the final results were adjusted by potential confounders. All analyses were performed using Empower (R) (www.empowerstats.com, Boston MA) and R (http://www.R-project.org). All P values were two-sided, and statistical significance was defined as *P*<0.05. Chi-square test analysis was performed only on complete cases, and logistic regression analysis was performed on patients without a single variable record as a group.

## Results

### Descriptive Analysis of Participants’ Characteristics

**Table 1** shows the characteristics of study participants. Of all 15,210 pregnancies included in this study, 7,789 (51.21%) resulted from fresh cycles and 7,421 (48.79%) were from frozen cycles. The mean maternal age was 29.94 ± 4.17 years, the mean male age was 30.80 ± 4.72 years, mean maternal BMI was 23.40 ± 3.56 kg/m^2^, and mean thickness of the endometrium was 10.36 ± 1.94 mm. Of all cycles, 8,740 (58%) cases were primary infertility and 6,630 cases (42.00%) were secondary infertility. There were 1,746 (11.48%) patients diagnosed with PCOS, 6,550 (43.15%) patients diagnosed with male factors, and 667 (5.17%) with uterine factors. Of all patients with male factors, 4,157 (63.47%) had mild-moderate oligosthenospermia (OAT), 1,707 (26.06%) had severe OAT, and 686 (10.47%) had obstructive azoospermia. The uterine factor details of study patients are shown in **Supplementary Table 1**. Of the 295 patients with congenital malformations, 1 case had a T-shaped uterus, 104 cases had a septate uterus, 37 cases had a bicorporeal uterus, and 153 cases had a hemi uterus. Two embryos were transferred in 12,979 (85.66%) cycles, while cleavage-stage embryos were transferred in 14,189 (95.43%) cycles.

**Table 1:** The basic characteristics of patients conceived through fresh/frozen ET cycles.

Characteristics	Value
Maternal age (years)	29.94 ± 4.17
Male age (years)	30.80 ± 4.72
Maternal BMI (kg/m^2^)	23.40 ± 3.56
Duration of infertility (years)	3.83 ± 2.80
Type of infertility	
Primary infertility, n (%)	8740 (58.00%)
Secondary infertility, n (%)	6330 (42.00%)
Infertility diagnosis, n (%)	
PCOS	1746 (11.48%)
Tubal factor	9126 (70.63%)
Male factors	6550 (43.15%)
Mild-moderate OAT	4157/6550 (63.47%)
Severe OAT	1707/6550 (26.06%)
Obstructive azoospermia	686/6550 (10.47%)
Endometriosis	586 (4.54%)
Unexplained factors	1628 (13.41%)
Uterine factors, n ( %)	667 (5.17%)
Previous spontaneous abortions, n (%)	
0	11203 (73.66%)
1	2764 (18.17%)
2	861 (5.66%)
≥3	381 (2.51%)
Thickness of endometrium (mm)	10.36 ± 1.94
Type of cycle, n (%)	
Fresh cycles	7789 (51.21%)
Frozen cycles	7421 (48.79%)
COS protocol	
Mid-luteal long	7789 (51.21%)
Early-follicular super-long	3612/7789 (46.37%)
GnRH antagonist	2495/7789 (32.03%)
Natural cycle	1057/7789 (13.57%)
Frozen-thawed protocol	
HRT	7421 (48.79%)
Down-regulation + HRT	5322 (71.72%)
Natural cycle	571 (7.69%)
Induced ovulation cycle	120 (1.61%)
Embryo stage, n (%)	
Cleavage embryo	14189/14868 (95.43%)
Blastocyst	679/14868 (4.57%)
No. of embryos transferred, n (%)	
1	716/15162 (4.72%)
2	12979/15162 (85.60%)
≥3	1467 /15162 (9.68%)

IVF, in vitro fertilization; ICSI, intra-cytoplasmic sperm injection; PCOS, polycystic ovary syndrome; OAT, Oligosthenospermia; COS, controlled ovarian stimulation; GnRH, gonadotropin-releasing hormone; HRT, hormone replacement therapy. Values are numbers (percentages) unless stated otherwise.

The total number of cycles varies among groups due to missing data.

**Figure 1:**
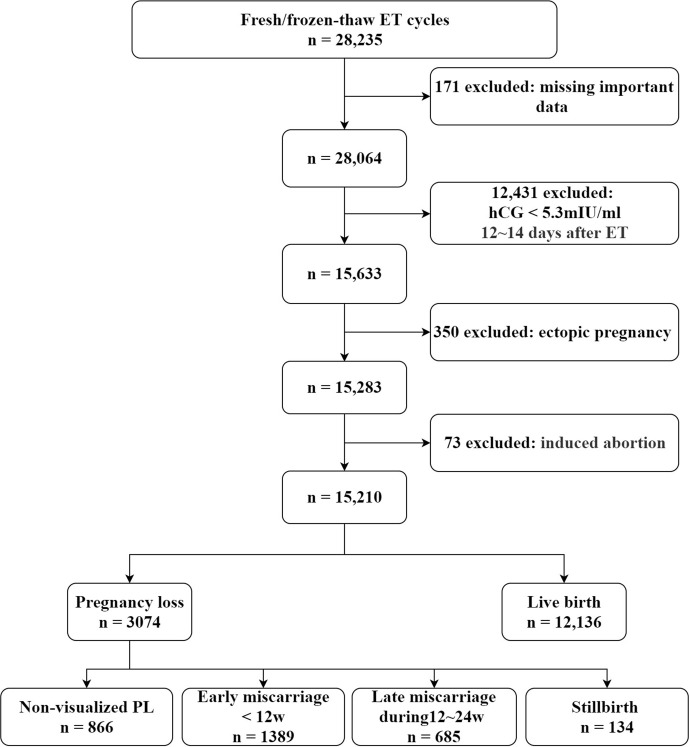
Flow chart for selection of patients from January 2014 to June 2019.

**Figure 1** is a flowchart showing a selection of the study participants and an overview of all the included fresh and frozen IVF/ICSI-ET cycles with corresponding pregnancy outcomes. Among a total of 28,235 fresh/frozen IVF/ICSI-ET cycles from January 2014 to June 2019, 12,341 cycles were excluded because of serum hCG < 5.3mlU/mL or a negative urine hCG test at 12~14 days after ET, and 171 cycles were excluded because of missing data. Another 350 cycles were excluded due to ectopic pregnancy, while 73 cycles were excluded due to medically induced abortion of the fetus with prenatally diagnosed structural or chromosomal abnormalities. After all exclusions, a total of 15,210 cycles were eligible for analysis, of which 12,136 ended in live birth and 3,074 ended in PL.

### Overall Pregnancy Outcomes

**Figure 2:**
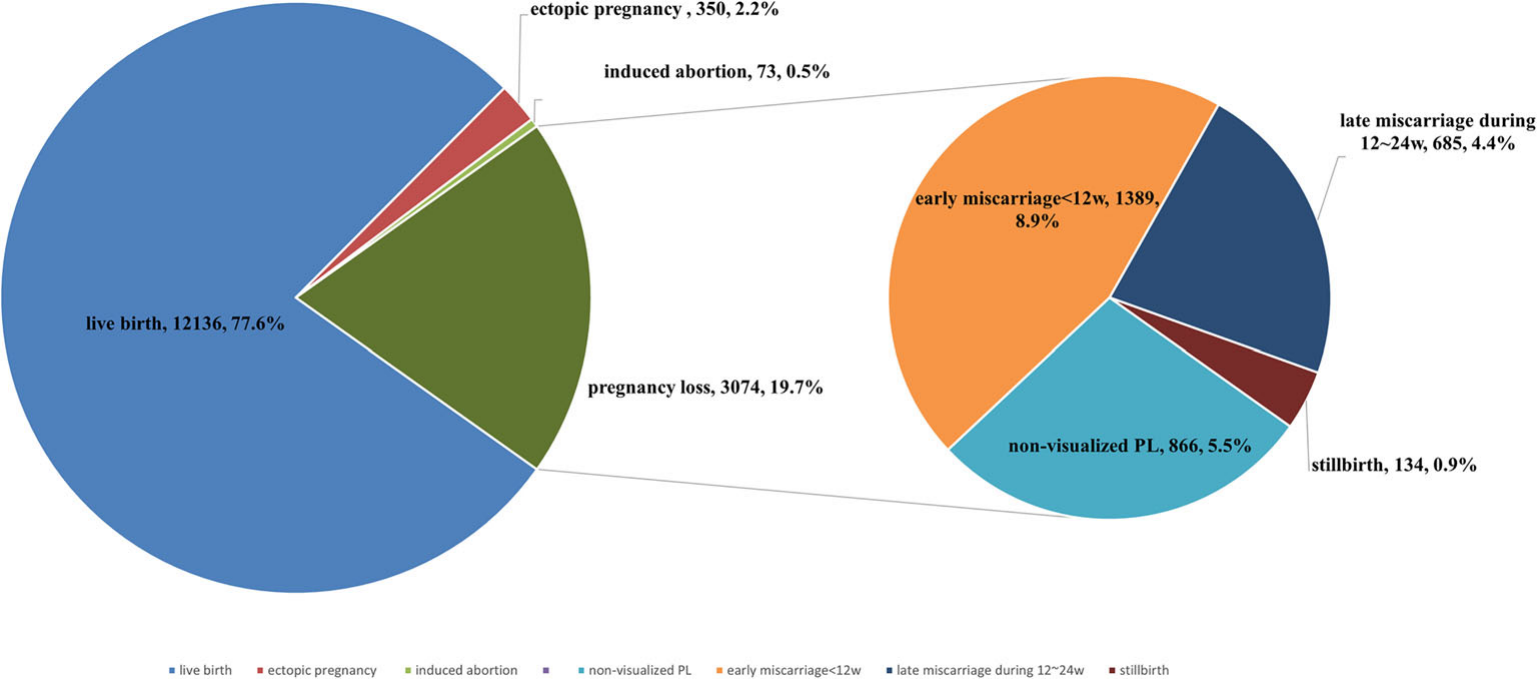
The proportion of live birth, pregnancy loss, induced abortion, ectopic pregnancy of all pregnancies. Percentage of non-visualized PL, early miscarriage, late miscarriage, and stillbirth of all pregnancy losses.

The following cycles were excluded: 350 (2.2%) cycles that resulted in ectopic pregnancy and 73 (0.5%) cycles that resulted in medically induced abortion. The pregnancy outcomes of all 15,210 included pregnancies conceived through ET are shown in **Figure 2**. The live birth rate of all pregnancies was 77.6%, and the PL rate was 19.7%. Of all 3,074 PL cycles, 866 (5.5%) ended in non-visualized PL, 1,389 (8.9%) ended in early miscarriage, 753 (4.4%) ended in late miscarriage, and 66 (0.9%) ended in stillbirth. The proportion diagram of the different types of pregnancy loss are visualized in **Figure 2**.

### PL Rates According to Patients’ Characteristics

PL rates according to patients’ baseline and treatment characteristics are shown in **Table 2**. Overall, PL rates varied among subgroups of couple’s age, maternal BMI, infertility diagnosis, COS and frozen-thawed protocol, no. of embryos transferred, thickness of the endometrium, and previous spontaneous abortions (SAs) (**Table 2**). Compared with primary infertility patients, secondary infertility patients demonstrated a significantly higher rate of PL (21.77% *vs* 19.85%, *P *= 0.0008). Patients with different male factors showed no difference on rate of PL (*P *= 0.167) (**Supplementary Table 2**). Higher PL rates were also observed in patients with frozen embryos and blastocysts transferred than those with fresh and cleavage-stage embryos transferred (21.59% *vs* 17.19%, *P* < 0.001 and 23.86% *vs* 19.99%, *P = 0.016* respectively). Our study also revealed that the PL rates decreased with the increase of endometrium thickness, and the difference was statistically significant (P = 0.0123). The PL rates showed no difference within the subgroups of uterine factors (**Supplementary Table 3**).

**Table 2:** Pregnancy loss rate based on baseline characteristics in fresh/frozen embryo transfer cycles from January 2014 to June 2019.

Characteristics	Pregnancy loss rate, n (%)	χ2	*P*-value
Maternal age(years)		198.44	<0.001
<35	2454 (18.83%)		
≥35, <40	466 (25.08%)		
≥40	154 (48.28%)		
Male age(years)		110.45	<0.001
<35	2303 (18.81%)		
≥35, <40	517 (23.46%)		
≥40	248 (33.51%)		
Maternal BMI (kg/m2)		42.49	<0.001
<18.5	122 (15.52%)		
≥18.5, <25	1742 (19.20%)		
≥25, <30	774 (22.22%)		
≥30	187 (26.49%)		
Type of infertility		6.99	0.008
Primary infertility	1742 (19.85%)		
Secondary infertility	1386 (21.77%)		
Infertility diagnosis treatment		72.97	<0.001
PCOS	416 (23.83%)		
Tubal factors	1889 (20.70%)		
Male factors	1252 (19.11%) 7		
Endometriosis	124 (21.16%)		
Unexplained infertility	309 (22.28%)		
Uterine factors	214 (34.5%)		
Previous SAs		12.99	0.011
0	2271 (20.18%)		
1	574 (20.65%)		
2	200 (23.04%)		
≥3	102 (26.70%)		
Type of cycle		28.79	<0.001
Fresh cycle	1339 (17.19%)		
Frozen cycle	1602 (21.59%)		
COS protocol		19.42	<0.001
Mid-luteal long	607 (16.80%)		
Early-follicular super-long	486 (19.48%)		
GnRH antagonist	234 (22.14%)		
Natural cycle	12 (26.09%)		
Frozen–thawed protocol		10.79	0.013
HRT	1210 (22.74%)		
Down-regulation + HRT	140 (24.52%)		
Natural cycle	32 (26.67%)		
Induced ovulation cycle	220 (19.00%)		
Stage of embryo		5.79	0.016
Cleavage stage	2836 (19.99%)		
Blastocyst	162 (23.86%)		
Thickness of endometrium(mm)		9.41	0.012
<7	41 (29.93%)		
7≤EM≤16	2843 (20.05%)		
>16	41 (29.93%)		
No. of embryos transferred		34.61	<0.001
1	202 (27.90%)		
2	2588 (19.85%)		
3	347 (23.59%)		

The categorial variables are presented as numbers (percentages within each group) and analyzed by using the χ2 test.

BMI, body mass index; IVF, in vitro fertilization; ICSI, intra-cytoplasmic sperm injection; PCOS, polycystic ovary syndrome; SA, spontaneous abortion; GnRH, gonadotropin-releasing hormone; COS, controlled ovarian stimulation; HRT, hormone replacement therapy.

### Association Between Risk Factors and PL

**Figure 3:**
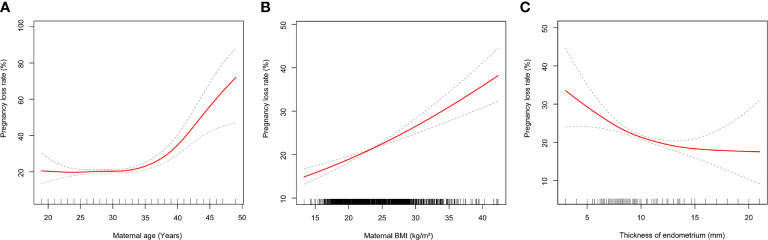
**(A)** Multivariate adjusted smoothing spline plots of pregnancy loss rate by maternal age. **(B)** Multivariate adjusted smoothing spline plots of pregnancy loss rate by maternal body mass index. **(C)** Multivariate adjusted smoothing spline plots of pregnancy loss rate by maternal thickness of endometrium. Solid red lines demonstrate the relationship between continues variables and pregnancy loss rate. Gray dotted curves represent the 95% of confidence intervals. Analyses were adjusted for number and stage of embryos transferred, duration of infertility, cycle type, maternal age, maternal body mass index, male age, indication for IVF treatment.

In univariate logistic regression analysis, shown in **Table 3**, the risk of PL increased with the couples’ age and maternal BMI increase. Secondary infertility was associated with higher risk of PL (OR 1.13, 95% CI 1.04-1.22). PCOS patients were associated with an increased risk of PL (OR 1.27, 95% CI 1.13-1.43), as were frozen embryos (OR 1.22, 95% CI 1.13-1.32) and blastocysts transfer (OR 1.26, 95% CI 1.05-.50). On the contrary, cases of PL were less likely to appear in patients with male factors (OR 0.85, 95% CI 0.78-0.92). Patients who had ≥2 embryos transferred were observed to have a lower risk of PL compared with those who had a single embryo transferred. In multivariate logistic analysis, shown in **Table 3**, maternal age remained as an independent risk factor for PL, but male age was no longer associated with PL after adjusting for the potential confounders. Compared with women under 35 years old, those between 35 and 40 had a higher risk of PL (OR 1.31, 95% CI 1.13-1.52), those aged over 40 had more than a three times higher risk of PL (OR 3.13, 95% CI 2.33-4.2) (**Table 3**). We further applied a two-piecewise linear regression model to examine the threshold effect of the maternal age on PL using a smoothing function (**Figure 3A**). The fitting curve showed that the PL rate was relatively low in women younger than 36 years, while in women aged over 36, the probability of PL increased by 22% with each additional year of age.

**Table 3:** Risk of pregnancy loss by univariate and multivariate logistic regression analysis.

Variable	Univariate analysis		Multivariate analysis	
	Crude OR (95% CI)	*P* value	Adjusted OR (95% CI)	*P *value
Maternal age(years)				
<35	Reference		Reference	
≥35, <40	1.47 (1.31 to 1.65)	<0.001	1.31 (1.13 to 1.52)	<0.001
≥40	3.99 (3.20 to 4.99)	<0.001	3.13 (2.33 to 4.20)	<0.001
Male age(years)				
<35	Reference		Reference	
≥35, <40	1.33 (1.19 to 1.48)	<0.001	1.07 (0.94 to 1.23)	0.312
≥40	2.16 (1.84 to2.53)	<0.001	1.20 (0.96 to 1.49)	0.102
Maternal BMI (kg/m2)				
<18.5	0.79 (0.65 to 0.96)	0.0173	0.81 (0.66 to 0.99)	0.0430
≥18.5, <25	Reference		Reference	
≥25, <30	1.20 (1.09 to 1.32)	<0.001	1.22 (1.10 to 1.34)	<0.001
≥30	1.52 (1.27 to 1.80)	<0.001	1.59 (1.32 to 1.90)	<0.001
Type of infertility				
Primary infertility	Reference		Reference	
Secondary infertility	1.13 (1.04 to1.22)	0.004	1.04 (0.93 to 1.16)	0.519
Infertility diagnosis				
PCOS	1.27 (1.13 to 1.43)	<0.001	1.21 (1.05 to 1.39)	0.007
Tubal factor	0.95 (0.87 to 1.04)	0.260	0.93 (0.82 to 1.07)	0.313
Male factor	0.85 (0.78, 0.92)	<0.001	0.94 (0.85, 1.05)	0.274
Endometriosis	1.02 (0.84 to 1.25)	0.826	1.03 (0.83 to 1.29)	0.776
Unexplained factors	1.10 (0.98 to 1.25)	0.118	0.97 (0.83 to 1.15)	0.746
Uterine factors	1.75 (1.48, 2.07)	<0.001	1.61 (1.29, 2.00)	<0.001
Previous SAs				
0	Reference		Reference	
1	1.03 (0.93 to 1.14)	0.548	0.94 (0.82 to 1.08)	0.384
2	1.18 (1.01 to 1.40)	0.043	0.97 (0.80 to 1.19)	0.784
≥3	1.44 (1.14 to 1.82)	0.0019	0.91 (0.69 to 1.21)	0.513
Type of cycle				
Fresh cycle	Reference		Reference	
Frozen cycle	1.22 (1.13 to1.32)	<0.001	1.12 (1.01 to 1.24)	0.027
COS protocol				
Mid-luteal long	Reference		Reference	
Early-follicular super-long	1.20 (1.05 to 1.37)	0.0074	1.11 (0.97 to 1.28)	0.1253
GnRH antagonist	1.41 (1.19 to 1.67)	<0.0001	1.16 (0.97 to 1.38)	0.1048
Natural cycle	1.86 (0.95 to 3.62)	0.0700	1.33 (0.65 to 2.75)	0.4332
Frozen–thawed protocol				
Natural cycle	1.0	1.0	1.0	
Down-regulation +HRT	0.89 (0.57 to 1.40)	0.620	0.94 (0.54 to 1.64)	0.821
HRT	0.81 (0.54 to 1.22)	0.311	0.90 (0.54 to 1.50)	0.692
Induced ovulation cycle	0.64 (0.42 to 0.99)	0.046	0.83 (0.49 to 1.41)	0.496
Stage of embryo				
Cleavage embryo	Reference		Reference	
Blastocyst	1.26 (1.05 to 1.50)	0.013	0.99 (0.78 to 1.24)	0.911
Thickness of endometrium(mm)	0.93 (0.92 to 0.95)		0.95 (0.92 to 0.97)	<0.0001
No. of embryos transferred				
1	Reference		Reference	
2	0.64 (0.54 to 0.76)	<0.001	0.70 (0.57 to 0.86)	<0.001
3	0.80 (0.65 to 0.98)	0.031	0.73 (0.57 to 0.94)	0.013

BMI, body mass index; PCOS, polycystic ovarian syndrome; HRT, hormone replacement therapy; EM, endometrium; SA, spontaneous abortion; COS, controlled ovarian stimulation; GnRH, gonadotropin-releasing hormone; HRT, hormone replacement therapy; OR, odds ratio; CI, confidence interval.

Results were calculated in each variable in the overall cycles, adjusted ORs were adjusted for all the other variables in the table using a binary logistic regression model. Data are presented as OR and 95% CI.

When analyzing all pregnancies, overweight and obese patients had significantly higher odds of PL compared to women with normal BMI (OR 1.22, 95% CI 1.10-1.34 and OR 1.59, 95% CI 1.32-1.90, respectively). However, a decreased risk of PL was observed in underweight women compared with normal weight women (OR 0.81, 95% CI 0.66- 0.99) (**Table 3**). The fitting curve for PL according to maternal BMI is shown in **Figure 3B**, it suggested that the risk of PL increased as the maternal BMI increased.

Type of infertility was no longer correlated to PL after adjustment. PCOS was an independent risk factor for PL (OR 1.21, 95% CI 1.05-1.39), the effect remained significant even after adjusting for potential confounders including maternal BMI (**Table 3**). However, patients with different male infertility diagnoses showed no difference on risk of PL (**Supplementary Table 4**). Uterine factors were related to the most significant increased risk of PL among other infertility diagnoses, with adjusted ORs ranging from 1.29-2.00 (**Table 3**). The association between uterine factor details and PL are shown in **Supplementary Table 5**. Patients with adenomyosis, septate, and hemi uteri demonstrated a higher risk of PL. However, tubal factors, endometriosis, and unexplained factors were not related to risk of PL as the multivariate analysis shows. Neither type of infertility (primary infertility and secondary infertility) nor previous SAs were associated with PL after adjustments. Notably, neither the COS protocol or frozen-thawed protocol was correlated with PL after adjustment (**Table 3**).

Thickness of the endometrium was negatively correlated with PL (OR 0.95, 95% CI 0.92- 0.97). In order to evaluate the non-liner relationship between thickness of the endometrium and PL rate, a fitting curve was applied (**Figure 3C**). The PL rate increased by 22% with each millimeter decline in endometrial thickness below 8 mm, but showed no difference in endometrial thickness above ≥8 mm. This association remained significant after adjusting for confounders. Women who had frozen embryos transferred were prone to a higher PL risk compared with those who had fresh embryos transferred (OR 1.22, 95% CI 1.13-1.32). However, the stage of embryos transferred no longer influenced PL after adjustment (**Table 3**). Our study also demonstrated that the risk of overall PL was lower in patients who transferred ≥2 embryos than patients who transferred a single embryo; adjusted ORs of PL ranged from 0.57-0.94 (**Table 3**).

### Risk Factors for Different Types of PL

We found that women aged ≥35 and <40 years were confronted with a higher risk of early miscarriage (OR 1.49, 95% CI 1.22-1.83). In addition to significantly higher odds of early miscarriage (OR 3.82, 95% CI 2.65-5.51), women aged ≥40 years also had an increased risk of non-visualized PL (OR 3.09, 95% CI 1.94-4.92) and late miscarriage (OR 2.79, 95% CI 1.64-4.77) (**Table 4**). The study suggested that being overweight was associated with non-visualized PL (OR 1.19, 95% CI 1.01-1.41) and late miscarriage (OR 1.38, 95% CI 1.16-1.65). Obesity was associated with an increased risk of early miscarriage (OR 1.47, 95% CI 1.14-1.91) and late miscarriage (OR 1.80, 95% CI 1.33-2.44). Notably, our results revealed higher odds of early and late miscarriage in the obesity cohort than the overweight cohort (**Table 4**). Furthermore, we found that PCOS was an independent risk factor for late miscarriage (OR 1.58, 95% CI 1.28-1.96), but not for non-visualized PL, early miscarriage, and stillbirth after adjusting for confounding variables (**Table 4**). Our results also demonstrated that patients with uterine factors were confronted with a higher risk of early miscarriage (OR 1.77, 95% CI 1.32- 2.38) and stillbirth (OR 3.29, 95% CI 1.58-6.85). As for the association of uterine factor details, adenomyosis showed a higher risk of non-visualized PL (OR 1.92, 95% CI 1.10-3.37), septate uterus and hemi uterus showed higher risk of early miscarriage (OR 1.81, 95% CI 1.01-3.23, OR 1.75, 95% CI 1.05-2.92, respectively) (**Supplementary Table 6**). Additionally, we observed that frozen embryo transfer was associated with a higher risk of early miscarriage (OR 1.35, 95% CI 1.19-1.53) and late miscarriage (OR 1.26, 95% CI 1.07-1.48), as shown in **Table 4**. Our results also suggested that patients who transferred ≥2 embryos had a lower risk of non-visualized PL and early miscarriage compared with those who transferred a single embryo, with an adjusted ORs range from 0.34-0.82. However, transferring three embryos appeared to be related to an increased risk of late miscarriage (OR 2.23, 95% CI 1.36 -3.66) (**Table 4**).

**Table 4:** Independent risk factors for different types of pregnancy loss.

Variable	Non-visualized PL	Early miscarriage	Late miscarriage	Stillbirth
	aOR (95% CI)	aOR (95% CI)	aOR (95% CI)	aOR (95% CI)
Maternal age(years)				
<35	Reference	Reference	Reference	Reference
≥35, <40	1.21 (0.93, 1.57)	**1.49 (1.22, 1.83)**	1.16 (0.87, 1.55)	1.33 (0.55,3.23)
≥40	**3.09 (1.94, 4.92)**	**3.82 (2.65, 5.51)**	**2.79 (1.64, 4.77)**	1.12(0.12,10.68)
Maternal BMI (kg/m^2^)				
<18.5	1.09 (0.79, 1.49)	0.82 (0.61, 1.10)	**0.50 (0.31, 0.82)**	_
≥18.5, <25	Reference	Reference	Reference	Reference
≥25, <30	**1.19 (1.01, 1.41)**	1.09 (0.94, 1.26)	**1.38 (1.16, 1.65)**	1.58 (0.88, 2.85)
≥30	1.19 (0.85, 1.67)	**1.47 (1.14, 1.91)**	**1.80 (1.33, 2.44)**	2.09 (0.80, 5.49)
Non-PCOS	Reference	Reference	Reference	Reference
PCOS	1.04 (0.83, 1.31)	1.14 (0.94, 1.37)	**1.58 (1.28, 1.96)**	1.31 (0.60, 2.87)
No-uterine factors	Reference	Reference	Reference	Reference
Uterine factors	1.44 (0.99, 2.08)	**1.77 (1.32, 2.38)**	1.24 (0.80, 1.94)	**3.29 (1.58, 6.85)**
Fresh cycle	Reference	Reference	Reference	Reference
Frozen cycle	1.04 (0.90, 1.21)	**1.35 (1.19, 1.53)**	**1.26 (1.07, 1.48)**	0.96 (0.56, 1.67)
Thickness of endometrium(mm)	**0.94 (0.89, 0.98)**	**0.94(0.91, 0.98)**	**0.94(0.89, 0.99)**	1.01(0.91, 1.13)
No. of embryos transferred				
1	Reference	Reference	Reference	Reference
2	**0.53 (0.40, 0.72)**	**0.63 (0.49, 0.82)**	1.37 (0.87, 2.16)	0.84 (0.19, 3.69)
3	**0.52 (0.36, 0.76)**	**0.47 (0.34, 0.65)**	**2.23 (1.36, 3.66)**	2.27(0.48,10.81)

PL, pregnancy loss; BMI body mass index; PCOS, polycystic ovarian syndrome; aOR, adjusted odds ratio. Results were calculated in each variable in the overall population, Data are presented as odds ratio (OR) and 95% confidence interval. Analyses were adjusted for type of infertility, duration of infertility, type of infertility, previous spontaneous abortions, stage of embryo, previous spontaneous abortions. Bold entries mean P-value was < 0.05 and there is statistically significant.

## Discussion

This retrospective cohort study showed that the overall PL rate was 19.7% and the live birth rate was 77.6% among 15,210 pregnancies conceived through fresh and frozen-thawed ET. We also found several maternal characteristics and IVF/ICSI cycle-specific factors, including maternal age, maternal BMI, thickness of the endometrium, uterine factors, frozen embryo transfer, and PCOS, associated with higher risk of PL. Besides, overweight, PCOS, and transferring three embryos were independent risk factors for late miscarriage. Women aged ≥40 years, obesity, frozen ET, and uterine factors were independent risk factors for early and late miscarriage. To our knowledge, this was the first retrospective cohort study to explore independent risk factors for PL throughout the entire pregnancy period and extensively distinguish risk factors for different types of PL.

Centers for Disease Control and Prevention (CDC) reported that PL rate among pregnancies conceived through ART was 29% in the United States between 1999-2002 (4), while, in 2016 and 2017, PL rates decreased to about 18% (26, 27). The incidence of PL in our study was 19.7%. One possible explanation for the reduction of PL is the development of luteal support and procedures of IVF/ICSI treatment. Several reports described a higher incidence of ectopic pregnancy after ART (2.2% to 4.5%) (28–30) than in spontaneous pregnancy. Remarkably similar to previous studies, our study demonstrated that the incidence of ectopic pregnancy in fresh and freeze-thaw ET was 2.2%.

Nowadays, more and more women delay childbearing for various reasons. However, maternal age was a risk factor for PL, which has been consistently reported and studied in previous studies (8, 31–33). As expected, the risk of miscarriage was strongly related to maternal age in our study. Compared with women under 35 years of age, the risk of PL increased to 25.98% in those between 35 and 40, and rose rapidly after 40 to 48.28%. The major underlying cause of these losses seemed to be chromosomal aneuploidy (31, 34) and decreased follicle reserves (35) in older women. So, women aged ≥36 years should be informed about their risk of PL before ART and close surveillance during pregnancy should be considered. Now pre-implantation genetic screening of embryos prior to transfer may reduce the probability of early PL resulting from aneuploidy, but safety and risks of the technology need further investigation.

The increasing occurrence of obesity associated with miscarriage has become a concern among reproductive-aged women (36). Our results showed that the adjusted odds ratios (ORs) with 95% confidence intervals (CIs) for PL were lower in cohorts with a low and normal maternal body mass index (BMI) and progressively increased with the increase of maternal BMI. Our study suggested that overweight patients have a higher risk of late miscarriage and obese patients have a higher risk of both early and late miscarriage. We may conclude that obesity had a more serious detrimental effect on reproductive outcomes than being overweight. Previous studies have also shown that obesity is a risk factor for early PL after IVF or ICSI (37, 38). Obesity may induce ovarian inflammation and reduce oocyte quality (39). The normal structure and function of the endometrium can also be negatively affected by obesity, as impaired stromal decidualization in obese women has been observed (39). Mediators would be insulin and leptin resistance, decreased glycodelin and insulin-growth factor binding protein 1(IGFBP-1), and increased plasminogen activator inhibitor 1, among others (40–43). This may explain subfecundity due to impaired endometrial receptivity, and may lead to placental abnormalities as manifested by higher rates of miscarriage, stillbirth, and preeclampsia in the obese population (44). Unlike prior studies (40, 45, 46), we found that underweight women were not associated with a higher risk of PL. On the contrary, our results showed a negative correlation between being underweight and late miscarriage. One possible explanation is that the small number of underweight patients in our study limited the power of conclusions. To our knowledge, there are very few studies focusing on the relationship between being underweight and late miscarriage. More research is warranted to ascertain the effect of being underweight on PL and the mechanisms leading to PL in women with increased BMI. Obese patients enrolled in assisted reproduction programs should be encouraged to lose weight before treatment, even moderate weight reduction might be beneficial (47–49).

Because of the close link between PCOS and obesity and the independent association of obesity with poor pregnancy outcomes, it is important to distinguish the possible confounding effect of BMI or other variables from that of PCOS (11). This study provided evidence to verify the effect of PCOS on PL with adjustment for BMI and several other confounding factors in a large cohort of women conceived through ET. We found that PCOS was an independent risk factor for PL, particularly for late miscarriage. Late miscarriage was so critical by any means, of which the risk factors could be very different from early miscarriage. PCOS is an independent risk factor for gestational diabetes and hypertensive disorders during the last two trimesters (50). We speculated that PCOS may cause more complications during the later phase of pregnancy, which may cause late miscarriage. The mechanism of high late miscarriage of PCOS may lie in metabolic changes of the endometrium, including glucose metabolism, hyperinsulinemia, and hyperandrogenemia, which may negatively affect endometrium function (51). A study showed that the placenta quality of PCOS patients was lower than that of normal patients, and the incidence of chorionic inflammation was significantly increased (52). Our findings did not corroborate the view that women with PCOS do not have a higher risk of first trimester PL (16) or do not have a higher risk after adjusting for fertility medication use (53, 54) or BMI (11). The definition of PL used varies among the above studies, which may explain the inconsistency of conclusions. Our study included embryo demise throughout the entire pregnancy and thus might better demonstrate the pregnancy outcome of PCOS patients. However, significant gaps in understanding how PCOS affects pregnancy still remain. This is most likely due to the complex, multifactorial etiology of PCOS, its range of potential confounders for pregnancy complications, and the variable methodology employed by different studies. This indicates that for patients with PCOS, more close surveillance is needed during 12~24 weeks of gestation. Additionally, more work should be done on the mechanism of PCOS, which led to the loss of pregnancy.

Many researchers have reported that there was a strong association between the thickness of the endometrium and pregnancy outcomes (3). K.E et al. found that PL rates increased with each millimeter decline in fresh cycles, when endometrial thickness was below 8 mm, and live birth rates declined as the endometrial thickness decreased below 7 mm in frozen ET cycles (55). Consistently, our results suggested that PL rates increased when the thickness of the endometrium was less than or equal to 8 mm, but there was no significant change when the endometrial thickness was above 8 mm. To the best of our knowledge, this was the first study which described a non-linear relationship between the thickness of the endometrium and PL rate. Similarly, Lan N et al. did not identify a statistically significant influence of endometrium thickness on live birth rates in either freeze-only or fresh embryo transfer in women with an endometrium thickness above 8 mm (56). However, Gallos et al. reported that the optimal endometrial thickness threshold of 10 mm or above maximized live births and minimized pregnancy losses (3). Endometrial thickness was measured during gonadotrophin stimulation and the maximum endometrial thickness before the trigger day was recorded in Gallos’ study. In our study, thickness of the endometrium on the oocyte maturation trigger day in fresh cycles and on the progesterone supplementation day in frozen cycles were recorded and subsequently used, which might be one possible reason for the different results. Furthermore, ultrasound measurements were inherently subjective even though the technique was standardized across the units.

Uterine factors were consistently associated with first and second trimester PL in addition to other obstetrical complications (16, 57), both in naturally and ART conceiving women. Uterine factors were associated with the most significant increased risk of PL among infertility diagnoses in our study. Initial evaluation of women enrolled in ART should include a uterine assessment such as three-dimensional transvaginal sonography or sonohysterography. Congenital uterine malformations such as uterine septum, intrauterine adhesions, and submucosal myomas should be managed surgically with operative hysteroscopy (58). However, for congenital uterine malformations other than a septate uterus, there is currently no high-quality evidence to support surgery for improving the live birth rate or decreasing the miscarriage rate (59). Moreover, more studies are warranted to further clarify the association between PL and uterine factors such as a T-shaped uterus, adenomyosis, and uterine myoma.

We found that the risk of non-visualized PL and early miscarriage decreased in patents who transferred more than one embryo than women who transferred one embryo. One possible explain is the incidence of multiple pregnancies tends to increase with the number of embryos transferred. If three fetal hearts were detected by ultrasound, only the loss of all fetuses can be recorded as PL. However, risk of late miscarriage was higher in patients who transferred three embryos in our study. Like previous studies reported, a high number of embryos transferred would result in high polyembryony (60). On average, twin pregnancies are delivered at 35.3 weeks of gestation with a mean birth weight of 2,336 g, twin pregnancies have an increased risk of preterm delivery (58.8% delivered before 37 weeks of gestation), cerebral palsy (7/1,000 live births), and infant mortality (23.6/1,000 live births) (61). As with all medical interventions, particularly elective treatments, the physicians’ aim is to minimize the risks associated with the procedure and outcome. In an effort to optimize PL and perinatal outcomes by reducing the risk of multiple gestations, it has become widely accepted that reproductive endocrinologists should aim to minimize the number of embryos transferred whenever feasible. We also found a statistically increased risk of PL in women who transferred frozen embryos, and several previous studies showed similar results (16, 31). Although FET is increasingly used for multiple indications nowadays, cautions are warranted for the alarmingly high PL risk.

Our results are strengthened by the large cohort of cycles and breadth of available patient and cycle characteristic data. We controlled for many factors that potentially affect PL. However, this study was limited by the availability of some data, we did not have data about the social and economic conditions which might have a significant impact on PL. Secondly, PCOS is a highly heterogeneous metabolic disorder, we did not further perform stratified analysis according to the metabolic index of these patients, such as fasting blood-glucose, fasting insulin, and serum lipid level, which might compromise pregnancy outcomes. Lastly, the relatively small number of stillbirths limited the ability of this study to assess the influencing factors of stillbirth.

## Conclusions

In summary, the risk factors associated with PL after fresh and frozen-thawed ET were identified through a large cohort study from China. The risk of PL increased with maternal age and maternal BMI increase. Overweight and obese women may benefit from weight loss programs. Since PCOS appeared to be associated with late miscarriage, both clinicians and patients should pay more attention to the risk of PL during 12~24 weeks of gestation. Surgical management of uterine factors before ART may be beneficial to improve pregnancy outcomes. Hopefully, the efficiency of the clinical evaluation of PL risk may be improved when the patients’ baseline and treatment characteristics are taken into account. Further research is needed to validate our results and investigate the mechanism underlying the reported associations.

## Data Availability Statement

The raw data supporting the conclusions of this article will be made available by the authors, without undue reservation.

## Ethics Statement

The studies involving human participants were reviewed and approved by the ethics committee of the Second Hospital of Hebei Medical University (Approval Letter NO. 2020-P052). The patients/participants provided their written informed consent to participate in this study.

## Author Contributions

A-MY and XX contributed equally to the paper. A-MY, XX, and XH had the idea for the study and designed the study. YH, A-MY, and XX did the data analysis. A-MY, XX, G-MH, NC, and YH provided statistical expertise. J-JW, Z-MZ and WW advised on the conduct and coordination of the study. A-MY wrote the first draft of the paper. A-MY, XX, G-MH and NC obtained funding. All authors contributed to the interpretation of the results and critical revision of the manuscript for important intellectual content and approved the final version of the manuscript. The corresponding author attests that all listed authors meet authorship criteria and that no others meeting the criteria have been omitted. XH is the guarantor.

## Funding

This study was supported by Medical Science Research Project of Hebei Province (20190496, 20211494), Natural Science Foundation of Hebei Province (H2019206707, H2019206674), Hebei Provincial Key Research Projects (20377714D, 21377721D), The National Key Research and Development Program (2018YFC1002104).

## Conflict of Interest

The authors declare that the research was conducted in the absence of any commercial or financial relationships that could be construed as a potential conflict of interest.
