# Supplementary material for: Risk Factors for Different Types of Pregnancy Losses: Analysis of 15,210 Pregnancies After Embryo Transfer
Source: Front Endocrinol (Lausanne). 2021 Jun 25;12:683236. doi: 10.3389/fendo.2021.683236 (PMC8267909; doi:10.3389/fendo.2021.683236)
Supplement: Supplementary file 1 [file DataSheet_1.docx]

Supplementary Table 1 Uterine factor details of the study population

| Characteristics | Value |
| --- | --- |
| Uterine factors, n ( %） | 667/12913 (5.17%) |
| Adenomyosis | 164/667 (24.59%) |
| Myoma | 148/667 (22.19%) |
| Intrauterine adhesions | 60/667 (9.00%) |
| Congenital Malformations | 295/667 (0.44%) |
| Dysmorphic uterus | 1/295 (0.03%) |
| Septate uterus | 104/295 (35.25%) |
| Bicorporeal uterus | 37/295 (12.54%) |
| Hemi uterus | 153/295 (51.86%) |

Classification for congenital uterus malformations were according to 2013

ESHRE/ESGE consensus on the classification of female genital tract congenital anomalies.

Supplementary Table 2 Pregnancy loss rate based on male factor details.

| Characteristics | Pregnancy loss rate, n (%) | χ2 | *P*-value |
| --- | --- | --- | --- |
| Mild-moderate OAT | 824 (19.82%) | 3.58 | 0.167 |
| Severe OAT | 308 (18.04%) |  |  |
| Obstructive azoospermia | 121 (17.64%) |  |  |

OAT: Oligosthenospermia

Supplementary Table 3 Pregnancy loss rate based on uterine factors

| Characteristics | Pregnancy loss rate, n (%) | χ2 | *P*-value |
| --- | --- | --- | --- |
| Adenomyosis | 60 (36.6%) | 3.03 | 0.39 |
| Myoma | 41 (27.7%) |  |  |
| Intrauterine adhesions | 21 (35.0%) |  |  |
| Congenital Malformations | 96 (32.5%) |  |  |
| Dysmorphic uterus | 1 |  |  |
| Septate uterus | 33 (31.7%) |  |  |
| Bicorporeal uterus | 14 (37.8%) |  |  |
| Hemi uterus | 48 (31.4%) |  |  |

Classification for congenital uterus malformations were according to 2013

ESHRE/ESGE consensus on the classification of female genital tract congenital anomalies.

Supplementary Table 4 The association between male factor details and pregnancy loss

| Variable | Univariate analysis | | Multivariate analysis | |
| --- | --- | --- | --- | --- |
|  | Crude OR (95% CI) | *P*value | Adjusted OR (95% CI) | *P*value |
| Non-male factors | Reference |  | Reference |  |
| Mild-moderate OAT | 0.85 (0.78 to 0.93) | 0.0003 | 0.98 (0.86 to 1.11) | 0.74 |
| Severe OAT | 0.98 (0.76 to 1.27) | 0.88 | 1.33 (0.92 to 1.92) | 0.13 |
| Obstructive azoospermia | 0.82 (0.67 to 1.00) | 0.05 | 0.92(0.60 to1.40) | 0.70 |

OAT: Oligosthenospermia, OR: odds ratio, CI: confidence interval. Analyses were adjusted for maternal age, male age, maternal BMI, duration of infertility, type of infertility, previous spontaneous abortions, type of cycle, stage of embryo, No. of embryo transferred.

Supplementary Table 5 The association between uterine factor details and pregnancy loss

| Variable | Univariate analysis | | Multivariate analysis | |
| --- | --- | --- | --- | --- |
|  | Crude OR (95% CI) | *P*value | Adjusted OR (95% CI) | *P*value |
| Non-uterine factors | Reference |  | Reference |  |
| Adenomyosis | 2.25 (1.63, 3.10) | <0.0001 | 1.76 (1.24, 2.50) | 0.0017 |
| Myoma | 1.48 (1.03, 2.13) | 0.0327 | 1.36 (0.92, 2.00) | 0.1217 |
| Intrauterine adhesions | 2.08 (1.22, 3.55) | 0.0068 | 1.82 (0.99, 3.33) | 0.0522 |
| Dysmorphic uterus | - |  | - |  |
| Septate uterus | 1.80 (1.19, 2.73) | 0.0054 | 1.70 (1.10, 2.64) | 0.0178 |
| Bicorporeal uterus | 2.35 (1.21, 4.58) | 0.0117 | 1.99 (0.99, 4.00) | 0.0544 |
| Hemi uterus | 1.77 (1.26, 2.50) | 0.0011 | 1.70 (1.17, 2.46) | 0.0050 |

OR: odds ratio, CI: confidence interval. Analyses were adjusted for maternal age, male age, maternal BMI, duration of infertility, type of infertility, previous spontaneous abortions, type of cycle, stage of embryo, No. of embryo transferred.

Supplementary Table 6 The association between uterine factor details and different kinds of pregnancy loss

|  | Non-visualized PL | Early miscarriage | Late miscarriage | Stillbirth |
| --- | --- | --- | --- | --- |
|  | OR (95% CI) | OR (95% CI) | OR (95% CI) | OR (95% CI) |
| Non-uterine factors | Reference |  | Reference |  |
| Adenomyosis | **1.92 (1.10, 3.37)** | 1.25 (0.74, 2.08) | 1.35 (0.66, 2.77) | 2.89 (0.84, 9.90) |
| Septate uterus | 1.53 (0.74, 3.16) | **1.81 (1.01, 3.23)** | 0.87 (0.31, 2.47) | 1.16 (0.15, 8.94) |
| Hemi uterus | 0.58 (0.24, 1.38) | **1.75 (1.05, 2.92)** | 1.20 (0.55, 2.61) | 1.75 (0.37, 8.17) |

PL: pregnancy loss, OR: odds ratio, CI: confidence interval. Analyses were adjusted for maternal age, male age, maternal BMI, duration of infertility, type of infertility, previous spontaneous abortions, type of cycle, stage of embryo, No. of embryo transferred.

Supplementary Table 7 The association between luteal support and pregnancy loss

|  | Univariate analysis | | Multivariate analysis | |
| --- | --- | --- | --- | --- |
|  | Crude OR (95% CI) | *P*value | Adjusted OR (95% CI) | *P*value |
| Dydrogesterone | Reference |  | Reference |  |
| Crinone and Dydrogesterone | 1.21 (1.04 to 1.41) | 0.01 | 1.07 (0.89 to 1.28) | 0.4758 |

OR: odds ratio, CI: confidence interval. Analyses were adjusted for maternal age, male age, maternal BMI, duration of infertility, type of infertility, previous spontaneous abortions, type of cycle, stage of embryo, No. of embryo transferred.
